# Supercritical Fluid Extract of *Putranjiva roxburghii* Wall. Seeds Mitigates Fertility Impairment in a Zebrafish Model

**DOI:** 10.3390/molecules26041020

**Published:** 2021-02-15

**Authors:** Acharya Balkrishna, Pradeep Nain, Monali Joshi, Lakshmipathi Khandrika, Anurag Varshney

**Affiliations:** 1 Drug Discovery and Development Division, Patanjali Research Institute, NH-58, Haridwar 249 405, Uttarakhand, India; pyp@divyayoga.com (A.B.); pradeep.nain@prft.co.in (P.N.); monali.joshi@prft.in (M.J.); lakshmipathi.khandrika@prft.in (L.K.); 2 Department of Allied and Applied Sciences, University of Patanjali, Patanjali Yog Peeth, Rorkee-Haridwar Road, Haridwar 249 405, Uttarakhand, India

**Keywords:** *Putranjiva roxburghii*, Putrajeevak seed oil, N-ethyl-N-nitrosourea mutagenesis, impaired fertility, zebrafish, β-sitosterol, follicle development, sperm motility

## Abstract

Putrajeevak (*Putranjiva roxburghii* Wall.; synonym *Drypetes roxburghii* (Wall.) Hurus) seeds have been used since ancient times in the treatment of infertility in the Ayurvedic system of medicine in India. In this study, the oil component of Putrajeevak seeds (PJSO) was extracted using the supercritical fluid extraction (SCFE) method using liquid CO_2_ and the constituents were analyzed using gas chromatography-flame ionized detectorand high-performance thin-layer chromatography. PJSO contained trace amounts of β-sitosterol with oleic and linoleic acids as the major fatty acid constituents. Male and female zebrafish were mutagenized with N-ethyl-N-nitrosourea (ENU) and fish that produced less than 20 viable embryos were selected for the study. SCFE oil extracts from the *P. roxburghii* seeds were used in this study to reverse fertility impairment. The mutant fish were fed with PJSO for a period of 14 days and the rates of fertility, conception, and fecundity were determined with wild-type healthy fish as a breeding partner. Treatment with PJSO increased the ovarian follicle count as well as the number of mature eggs, while reducing the number of ovarian cysts. Sperm count as well as sperm motility were greatly enhanced in the ENU-mutagenized male zebrafish when treated with PJSO. The results obtained in this study demonstrate the effectiveness of *P. roxburghii* seed oil in reversing impaired fertility in both male and female zebrafish models.

## 1. Introduction

Putrajeevak (*Putranjiva roxburghii* Wall.; synonym *Drypetes roxburghii* (Wall.) Hurus) seeds have been used in the traditional Indian system of Ayurveda since ancient times for the treatment of infertility in both males and females. Many classical medicinal texts such as Candra-Nighaṇṭu (10th Century A.D.), Nighaṇṭuśeṣa (12th Century A.D.), Saraswatī Nighaṇṭu(16th Century A.D.), and others have an extensive discussion on the use of *P. roxburghii* in alleviating infertility. The plant belongs to the Euphorbiaceae family and is native to Southeast Asia, the Indian subcontinent, Japan, southern China, and New Guinea. The deseeded and powdered fruit is used against cough, cold, and celiac disease [1]. Its antipyretic and anti-inflammatory properties are thought to be useful in fertility and gynecological ailments [2]. Several different phytochemicals have been identified from *P. roxburghii* root, leaf, and stem including glycosides, saponins, triterpenes, and flavonoids.

Though the seeds are consumed, not much is known about the fatty acids and other constituents of the oil present in the seeds. This is the first time that the fatty acids and phytosterol constituents of *P. roxburghii* seeds were extracted and identified. The supercritical fluid (SCF) extraction method for oils is an environmentally safe technique and has several advantages over traditional extraction processes [3]. Compared to the traditional solvent-based extraction system, SCF extraction is more robust due to less variations from batch to batch. Supercritical fluids under pressure have a higher diffusivity compared to normal solvents and therefore can penetrate porous solid materials more effectively [4]. The use of food-grade liquid carbon dioxide in the extraction process at very mild temperatures protects the heat sensitive constituents from degradation and increases their yield in the final extract. However, one of the drawbacks of using CO_2_ as a solvent is that it is non-polar, and therefore, extraction of certain polar compounds may be suboptimal [5].

In our study, the oil from *P. roxburghii* (Putrajeevak) seeds extracted using the supercritical fluid extraction process was analyzed for its constituent fatty acids and sterols. The oil (Putrajeevak Seed Oil, PJSO) was then used for assessing its properties in reversing the fertility impairment seen in N-ethyl-N-nitrosourea (ENU) mutagenized zebrafish. Zebrafish are versatile as a model organism due to their high reproductive rate and fecundity, and being vertebrates, they can be easily correlated with higher vertebrates. The causes for infertility are numerous and can vary from ovarian defects such as polycystic ovary syndrome, a diminished ovarian reserve of eggs that may be related to aging, and the presence of immature eggs. Though the underlying causes of such defects are not known, it can either be genetic or lifestyle-related. In males, the cause of infertility could generally be due to low sperm count or genetic disorders that can lead to lower sperm production or immotile sperm. ENU mutagenesis is a widely accepted method to induce random point mutations [6] that can be reversed and the method can be easily modified to reduce the mortality that is generally seen in such mutagenesis methods. The mutants that impact the fertility seem to target spermatogenesis in males, while altering the ovarian morphology and cytology in females [7].

In the current study, we have used ENU mutagenesis to generate fertility impairment mutations in both females and males. The impaired mutants that generated less than 20 embryos were selected for treatment with the Putrajeevak seed oil. Letrozole, an aromatase inhibitor, was used as a standard reference drug for the female infertility study group. Letrozole has been shown to have a better number of live births in women with polycystic ovary syndrome [8]. Clomiphene is an anti-estrogen thought to increase the quality of sperm in cases of male infertility [9] and was used as a reference drug for the male infertility study.

The study was aimed at not only understanding the phytochemical composition of the supercritical fluid extract of the seeds but also to substantiate the traditional Ayurvedic knowledge of the use of the seeds to restore fertility. We have demonstrated the recovery of fertility, conception, and fecundity rates in fertility-impaired zebrafish upon treatment with Putrajeevak seed oil. We also show the recovery of ovarian and testicular cytology upon treatment.

## 2. Results

### 2.1. Fatty Acid Content in Supercritical Fluid Extract of Putrajeevak Seeds

Several batches of extraction were performed and an average yield of 3% oil was obtained. The batch of oil used for these studies was obtained from powdered seeds (2 kg) of Putrajeevak (*Putranjiva roxburghii* Wall.) using a supercritical CO_2_ extraction procedure to obtain 60 gm of the oil extract (PJSO) with a yield of 3%. For identification and quantification of the fatty acid constituents present in the oil extract, the AOCS official method, Ce 2-66, was used to produce fatty acid methyl esters (FAME). The mixture from the PJSO was compared with a standard mixture (37 FAMEs) using GC-FID. The chromatography analysis identified four fatty acids present in high quantities and two others in relatively lower amounts (Table 1). The other 31 fatty acids, according to the standard mix, were below the quantitation limits of the assay.

### 2.2. PJSO Contains Trace Amounts of β-Sitosterol

**Figure 1:**
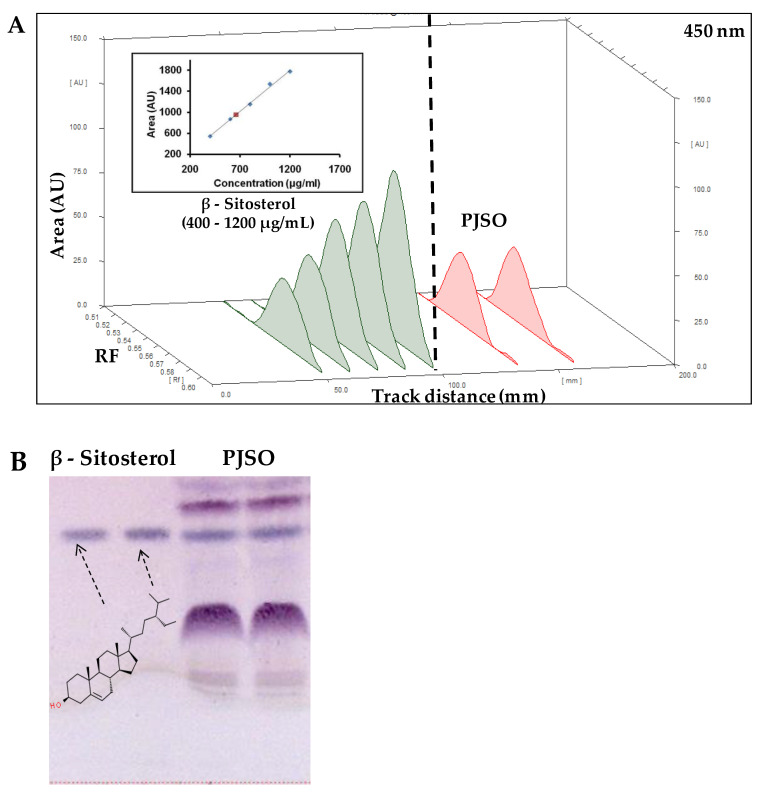

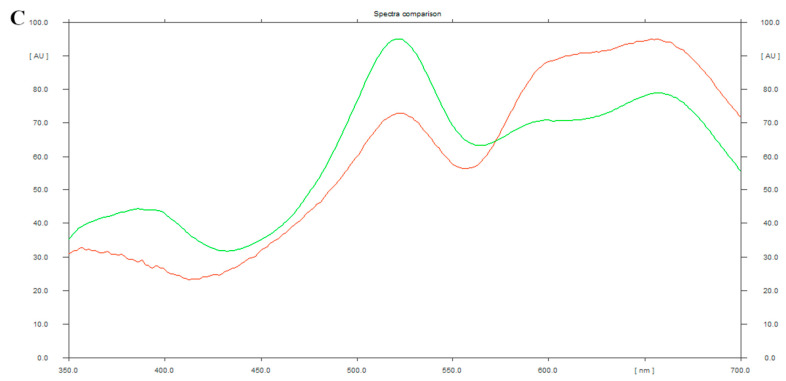
High-performance thin layer chromatography of *Putranjiva roxburghii s*eed oil (PJSO) supercritical fluid extract. (**A**) Chromatogram showing spectral peaks and area under the curve model for known concentrations of β-sitosterol standard (green lines) and PJSO (red lines) at 450 nm. Inset shows the linearity curve of standard at various concentrations. (**B**) Representative photograph of TLC plate developed after derivatization with anisaldehyde sulfuric acid reagent under white light. The concentrations of standard β-sitosterol were 1000 and 1200 µg/mL. The injection volume of PJSO was 1 µL. (**C**) Overlay spectral scans of the developed bands on a representative TLC plate at 350 to 700 nm range. β-sitosterol standard is represented by the green line and β-sitosterol from the PJSO sample is shown in red.

High-performance thin layer chromatography (HPTLC) analysis of PJSO was performed with chloroform:methanol (9:1) as the mobile phase (Figure 1A). After derivatization with the Anisaldehyde Sulfuric acid reagent (Figure 1B), a spectral scan (350 to 700 nm) was performed to detect β-sitosterol along with a standard solution of known concentration (Figure 1C). The standard curve showed good linearity with 400 to 1200 ng of the standard. The band intensity obtained by densitometric analysis was plotted against the standard curve to derive the concentration of β-sitosterol in the PJSO sample. The amount was calculated to be 0.98% in the supercritical fluid extract of Putrajeevak seeds.

### 2.3. P. roxburghii Seed Oil Increases the Number of Positive Spawning Events in the Fertility-Impaired Female and Male Zebrafish

Fertility impairment was induced in the zebrafish using ENU mutagenesis as described in the methods section. Female fish that produced less than 20 embryos when used in breeding events with healthy males and vice versa were used in the study. A total of 550 females were exposed to the ENU mutagenesis protocol, out of which 542 survived. The fish were rested for 14 days, after which the fertility rate was assessed over two ovulation cycles. The number of female mutagenized fish with fertility impairment as judged by those producing less than 20 embryos was 413. Treatment with ENU followed by metronidazole in the male fish led to 519 survivors and 450 males that produced less than 20 embryos when mated with healthy females.

**Figure 2:**
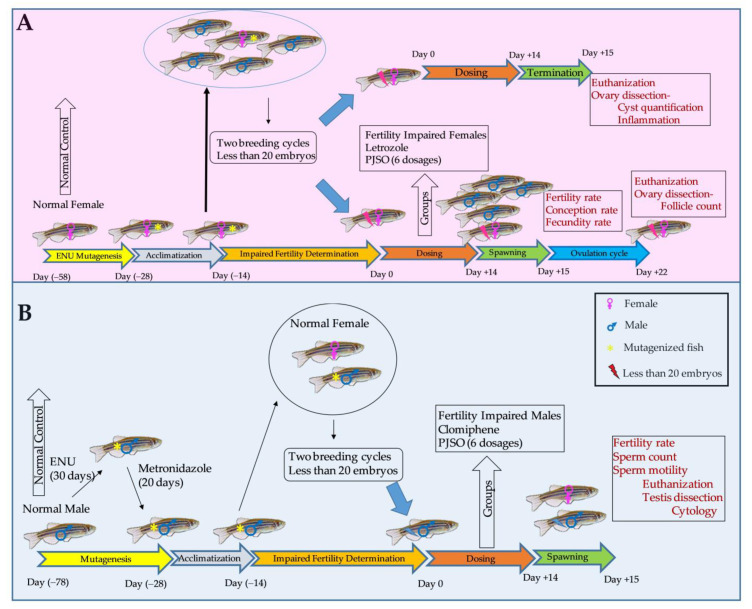
Schematic representation of the design, timelines, and endpoints for the study. (**A**) Study design for the fertility-impaired female fish. (**B**) Fertility-impaired male fish. The dosage used for the standard drug letrozole was 0.036 µg/kg/day in the female zebrafish and clomiphene at 0.36 µg/kg/day in the males. The dosages used for the *P. roxburghii* seed oil (PJSO) were 0.2, 0.5, 1, 5, 10, and 100 µg/kg/day.

Once the required number of mutagenized fish were randomized into eight groups with 24 fish each, they were treated with human relevant dosage of letrozole (reference drug for females) or clomiphene (reference drug for males) and six different dosages of the PJSO. A schematic of the study is shown in Figure 2.

Throughout the 14-day dosing period, the fish in the different groups were monitored for mortality each day. Along with the control group, the fertility-impaired group, the group treated with letrozole, and the different *P. roxburghii* seed oil-treated groups did not show any mortality and were 100% viable.

The number of positive spawning events that resulted in the formation of embryos was recorded and expressed as a percentage. The female healthy control group had 100% spawning events, while this was just 83.3% (Table 2). Treatment with either letrozole or PJSO restored the number of positive spawning events to 100%.

The control males showed a positive spawning rate of 100%, with all 24 breeding pairs producing embryos. The fertility-impaired group showed a lower positive spawning rate of 75%, with only 18 males producing viable spermatozoa to produce embryos (Table 2). Treatment with clomiphene showed a slightly better positive spawning rate at 88%, with 21 breeding pairs showing positive spawning. This increase was statistically significant (*p* < 0.0001) when compared to the fertility-impaired group. The different dosages of PJSO showed a dose-dependent increase, with the 0.2 µg/kg/day dosage showing only a modest increase with sperm from 19 males (79.1%) successfully forming embryos. Treatment with 0.5 µg/kg/day PJSO showed a positive spawning rate comparable to that of clomiphene at 91.6% (22 of 24 males). PJSO at 1 µg/kg/day had 23 with positive spawning (95.8%), while 5, 10, and 100 µg/kg/day showed complete recovery of the fertility impairment.

### 2.4. Putrajeevak Seed Oil Increases the Fertility and Fecundity in Fertility-Impaired Zebrafish Model

Female zebrafish showing impaired fertility were treated with letrozole (0.036 µg/kg/day) as a reference standard. Feed mixed with Putrajeevak seed oil (PJSO) at various concentrations (0.2, 0.5, 1, 5, 10, and 100 µg/kg/day) for 14 days was compared to the healthy control fish and the fertility-impaired model fish on the 15th day, to assess the recovery of fertility.

**Figure 3:**
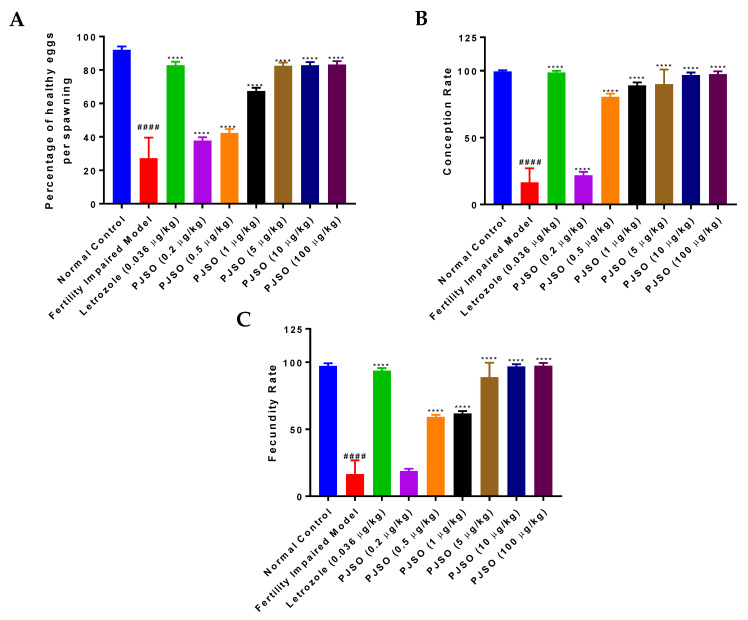
Treatment with PJSO reversed fertility impairment in female zebrafish. (**A**) Percentage of healthy eggs per spawning was calculated from different study groups. (**B**) Conception rate was calculated by the number of spawning events that formed a viable embryo to the total number of spawning events. (**C**) The number of spawning events that led to the birth of a viable larvae from embryos to the total number of successful spawning events was calculated as the fecundity rate. Total number of breeding pairs were 24 (*n* = 24). Data are represented as mean ± SD and one-way ANOVA followed by Tukey’s multiple comparison test was used to arrive at statistical significance; #### represents *p* < 0.0001 compared to the healthy control, **** represents *p* < 0.0001 compared to the fertility-impaired group.

Female mutant fish were allowed to spawn with healthy males and the percentage of healthy eggs was assessed per spawning. Healthy control fish showed a higher percentage of healthy eggs per spawning (92%) and this was significantly reduced (*p* < 0.0001) in the fertility-impaired group with only 27% of healthy eggs (Figure 3A). Letrozole restored the healthy egg percentage to about 83%, which was significantly higher (*p* < 0.0001) than that of the fertility-impaired group. Treatment with PJSO showed a gradual and dose-dependent increase in the percentage of healthy eggs, and was significantly higher (*p* < 0.0001) than the fertility-impaired group, even with the lowest dosage of 0.2 µg/kg/day. This dosage was calculated to be 1/5th of the daily recommended dosage prescribed in humans. The human equivalent dosage per day restored the percentage of healthy eggs to about 65%, while the groups treated with higher dosages of PJSO had healthy egg percentages equivalent to that seen with letrozole at more than 82%.

The rate of conception is calculated as the number of viable embryos to the total number of positive breeding events. In the healthy control fish, the rate of conception was observed to be 99.5% and this was drastically reduced to about 16% in the fertility-impaired group (Figure 3B). When compared to the fertility-impaired group, treatment with letrozole showed a highly significant increase (*p* < 0.0001) in the rate of conception at 98.6%. The lowest dosage of PJSO showed only a slightly significant increase (*p* < 0.05) in the conception rate at about 21%. At 0.5 µg/kg/day and higher, there was a highly significant increase in the conception rate, which was restored to levels comparable to the healthy control group at 10 µg/kg/day (96.8%) and 100 µg/kg/day (97.4%).

Of the healthy and viable embryos, the ones that survived to become larvae at 10 dpf were used to calculate the fecundity rate based on the total number of fertile embryos. The healthy control group showed a fecundity rate of 97.3%, while the fertility-impaired group showed a highly significant reduction (*p* < 0.0001) at just 16.4% (Figure 3C). Interestingly, the lowest dosage of PJSO showed only a modest and non-significant increase in the fecundity at 18.8%. Higher dosages showed a dose-dependent highly significant increase (*p* < 0.0001), with the 10× (10 µg/kg/day) and 100× (100 µg/kg/day) dosages showing recovery of the fecundity rate to near normal levels, at 96.8% and 97.4%, respectively. In comparison, treatment with letrozole showed a slightly lower rate (93.9%), which was still highly significant (*p* < 0.0001) when compared to the fertility-impaired group.

### 2.5. Treatment with Putrajeevak Seed Oil Showed Increased Egg Reserve and Recovery of Anatomical Structures in the Ovary

The reproductive potential of any ovary is measured by the ovarian reserve of eggs, dependent upon both by the quality and quantity of eggs present in the growing oocytes during an ovulation cycle. A diminished ovarian reserve represents a loss of normal reproductive potential in the ovaries. Intact ovaries from all the fish in each group were gently dissected out and the follicles containing eggs in various stages of development were teased out onto a counting slide.

The ovarian reserve at each stage of egg development was compared across the different groups in the study (Table 3). The fertility-impaired group showed a significant reduction (*p* < 0.0001) in the number of follicles at the various stages of egg development such as Primary growth (PG), Pre-vitellogenic (PV), Early vitellogenic (EV), Mid-vitellogenic (MV), and Full growth (FG). In comparison, treatment with letrozole showed a significant increase (*p* < 0.0001) in the number of follicles at each developmental stage compared to the fertility-impaired model. When the total number of follicles was taken into account, the ovarian egg reserve was lower than that seen in the healthy control fish but was still significantly higher than that seen in the fertility-impaired group (Table 3). Treatment with the lowest dosage (0.2 µg/kg/day) of PJSO had a modest but not significant increase in the PG, PV, and MV stages but showed a slightly significant increase (*p* < 0.05) in the EV stage and a highly significant increase (*p* < 0.0001) in the FG stage eggs. Overall, there was a highly significant increase (*p* < 0.0001) in the total number of ovarian egg reserves in this treatment group. The group of fish treated with PSJO at 0.5 µg/kg/day showed a significant increase (*p* < 0.0001) in the follicle count at all the stages of egg development except for the Mid-vitellogenic stage (*p* < 0.05), though the overall egg count was significantly higher than the fertility-impaired group. The total number of egg reserves showed a dose-dependent increase with an increasing dose of PJSO (Table 3). The total egg count in the 100 µg/kg/day treatment group was higher than even the letrozole-treated group, suggesting a recovery from the dysfunctional ovary seen in the fertility-impaired group.

**Figure 4:**
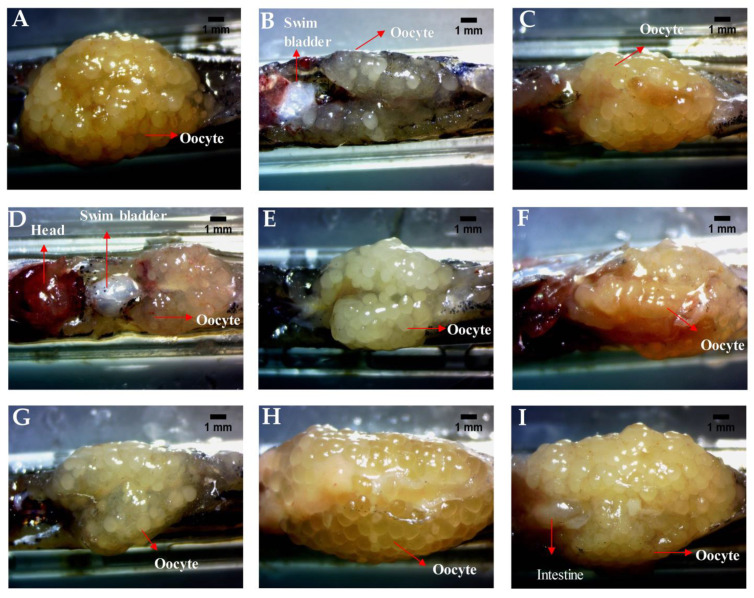
PJSO induced recovery of ovarian morphology in fertility-impaired female zebrafish. Representative images from the various study groups were taken with a stereomicroscope under 10× objective magnification. (**A**) Healthy control, (**B**) Fertility-impaired group, (**C**) Fertility-impaired fish treated with 0.036 µg/kg/day letrozole, (**D**) Fertility-impaired females treated with 0.2 µg/kg/day PJSO, (**E**) Treatment group with 0.5 µg/kg/day PJSO, (**F**) Treatment group with 1 µg/kg/day PJSO, (**G**) Treatment group with 5 µg/kg/day PJSO, (**H**) Treatment group with 10 µg/kg/day PJSO, and (**I**) Treatment group with 100 µg/kg/day PJSO. There were no outliers in each of the study groups and minor or negligible amounts of variation were seen in all ovaries from each group.

**Figure 5:**
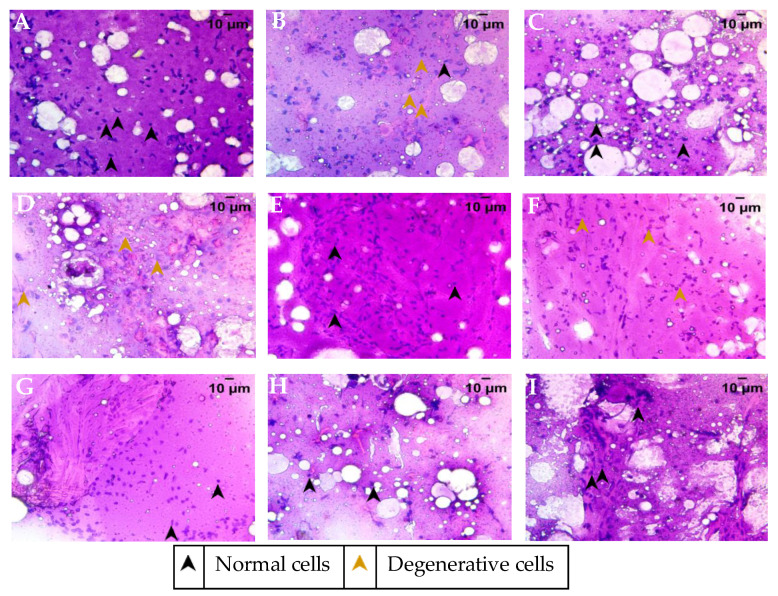
Cytology smear of ovary from the various treatment groups stained with Hematoxylin–Eosin and representative images were captured at 40× objective magnification. (**A**) Healthy control group, (**B**) Fertility-impaired group, (**C**) Letrozole treatment group (0.036 µg/kg/day), (**D**) Fertility-impaired females treated with 0.2 µg/kg/day PJSO, (**E**) Treatment group with 0.5 µg/kg/day PJSO, (**F**) PJSO (1 µg/kg/day) treatment group, (**G**) PJSO (5 µg/kg/day) treatment group, (**H**) PJSO (10 µg/kg/day) treatment group, and (**I**) Treatment group with 100 µg/kg/day PJSO.

In a parallel experiment, the fish dosed similarly were used for dissecting the ovary for overall morphology and cytological examination. The ovary was dissected and observed under the 10× bright field objective of a stereomicroscope to study the general size and health of the ovary. The healthy individuals showed an intact ovary with a high volume of eggs in various stages of development (Figure 4A) and there were no visible signs of hemorrhage. The ovary dissected from the fertility-impaired group was less voluminous with a lower number of follicles and most of them were premature (Figure 4B). A few follicular cysts were also identified by their opaqueness; other whitish fluid-filled follicles having a very thin outer follicular epithelium that ruptures during handling were also observed. The presence of these abnormalities showed that the follicles have not matured properly in the fertility-impaired group. Ovaries from all the fish from each group were dissected out and there were very minor or negligible amounts of variations in overall appearance. There were no overt differences noticed possibly due to the selection criteria used for identifying fertility impairment in the fish. Treatment with letrozole showed well-defined follicles in various stages of development and the ovary was voluminous when compared to the fertility-impaired group (Figure 4C). PJSO at lower dosages, 0.2 µg/kg/day (Figure 4D) and 0.5 µg/kg/day (Figure 4E), did not show much improvement in the gross anatomy of the ovary, with a less voluminous appearance as well as loosely packed follicles within the ovary sac. The follicles appeared to be covered in a very thin outer layer of parenchyma housing premature follicles. Treatment with medium dosages showed a gradual increase in the follicular count, with the 1 µg/kg/day (Figure 4F) and 5 µg/kg/day (Figure 4G) showing a slightly better follicular distribution. The high dosages of 10 µg/kg/day (Figure 4H) and 100 µg/kg/day (Figure 4I) showed a better dose-dependent recovery of the ovarian morphology as seen by an enriched follicle count at various stages of development.

Cytology smears of the ovary were stained with Hematoxylin and Eosin for determination of the cellular distribution and overall health of the ovary. Ovarian smear from healthy fish showed a well-defined nuclear staining and an eosinophilic cytoplasm with a high distribution of follicular epithelium and supportive parenchyma (Figure 5A). The smear showed a good distribution of densely stained cells throughout. The fertility-impaired group showed a degenerative nucleus of the follicular epithelium (Figure 5B). The cytoplasm was fluidic and, together with the degenerative nucleus, suggested the cytological features of a cyst rather than a healthy follicular epithelium, which was much reduced in its distribution. Overall, the cytological smear from the fertility-impaired group showed a higher presence of cysts rather than follicles. Treatment with letrozole restored most of the damage seen with fertility impairment, with visible yolk vesicles that are slightly eosinophilic (Figure 5C). The cytological smear showed the presence of a number of post-vitellogenic follicles, suggesting a mature developmental stage of the ovary. PJSO at 0.2 µg/kg/day (Figure 5D) showed a higher presence of degenerative nuclei of the follicular epithelium. The cytoplasmic staining was more basophilic, suggesting that the dosage was not enough to recover fertility impairment. Treatment with 0.5 µg/kg/day PJSO showed the presence of a few well-defined follicles with follicular epithelium and supporting parenchyma (Figure 5E). A few eosinophilic yolk vesicles were also seen in the cytology smear, suggesting that a few matured follicles were also present. This suggests that the dosage was starting to show recovery though was not enough to restore the damage completely. The cytology smear from 1 µg/kg/day showed largely basophilic cells, suggesting that most of the follicles in this group are at a pre-vitellogenic stage of growth (Figure 5F). Treatment with higher dosages at 5 µg/kg/day (Figure 5G), 10 µg/kg/day (Figure 5H), and 100 µg/kg/day (Figure 5I) showed a well-defined nucleus present in the follicular epithelium of the cytology smears. A good distribution of eosinophilic yolk vesicles was noted in a dose-dependent manner, suggesting a rescue of the cytological features in parallel with that seen in the gross anatomy of the ovary.

### 2.6. Putrajeevak Seed Oil Reversed Ovarian Cyst Formation and Pelvic Inflammation

**Figure 6:**
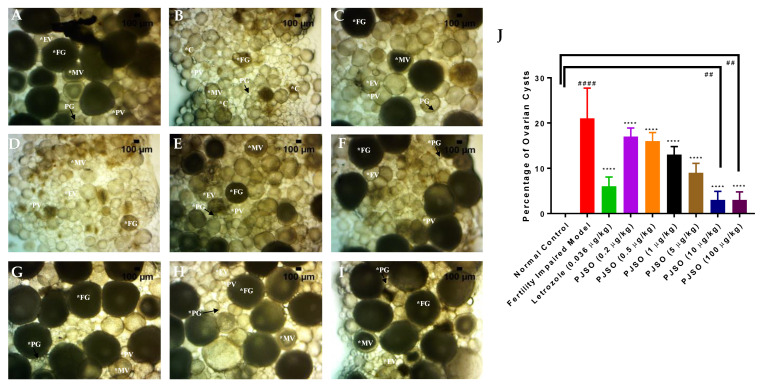
Identification and quantification of ovarian cysts in the ovary of the various study groups. Ovaries were imaged under the 40× objective of a stereomicroscope and the number of cysts was quantified (C—cyst; PG—Primary growth; PV—Pre-vitellogenic; EV—Early vitellogenic; MV—Mid-vitellogenic; FG—Full growth). Representative images from (**A**) Healthy control group, (**B**) Fertility-impaired group, (**C**) Treatment group with 0.036 µg/kg/day letrozole, (**D**) Fertility-impaired female zebrafish treated with 0.2 µg/kg/day PJSO, (**E**) 0.5 µg/kg/day PJSO treatment group, (**F**) 1 µg/kg/day PJSO, (**G**) 5 µg/kg/day PJSO, (**H**) 10 µg/kg/day PJSO, and (**I**) 100 µg/kg/day PJSO treatment group. (**J**) Data are represented as mean ± SD and one-way ANOVA followed by Tukey’s multiple comparisons test was used to determine statistical significance. ## represents *p* < 0.01 compared to the healthy control group, #### represents *p* < 0.0001 compared to the healthy control group, **** represents *p* < 0.0001 compared to the fertility-impaired group, *n* = 24.

A dissected ovary from each study group was observed under a bright field 40× objective to identify the presence of cysts in the ovary. They are identified as darker colored follicles without the presence of yolk in the cells. The ovary from the healthy control showed the presence of yolk-filled follicles at various developmental stages from the primary growth to fully mature eggs. The follicle stages were identified by their morphological features under a bright field microscope, including size and color. There was no evidence of cysts seen in this group (Figure 6A). The fertility-impaired group showed the presence of fluid-filled cells with degenerative nuclei that lack yolk (Figure 6B). This group showed a significant increase (*p* < 0.0001) in the number of cysts compared to the healthy control group (Figure 6J). Treatment with letrozole showed a healthy ovary with a higher number of follicles in the full growth stage of development (Figure 6C). In addition, there was a significant reduction (*p* < 0.0001) in the number of cysts present. Treatment with PJSO showed a dose-dependent reduction in the number of cysts. Treatment with low and medium dosages of PJSO at 0.2 µg/kg/day (Figure 6D), 0.5 µg/kg/day (Figure 6E), 1 µg/kg/day (Figure 6F), and 5 µg/kg/day (Figure 6G) still showed the presence of a good number of cysts. Though this number was significantly lower (*p* < 0.0001) than that seen with in the fertility-impaired group, it was still highly statistically significant (*p* < 0.0001) when compared to the healthy control group and was higher than that of the letrozole-treated group. Treatment with high dosages of PJSO at 10 µg/kg/day (Figure 6H) and at 100 µg/kg/day (Figure 6I) showed a significant reduction in the number of cysts compared to the fertility-impaired group. This was even lower than that seen with the letrozole-treated group but was still significantly higher (*p* < 0.01) than that of the healthy control group where no cysts were identified (Figure 6J).

**Figure 7:**
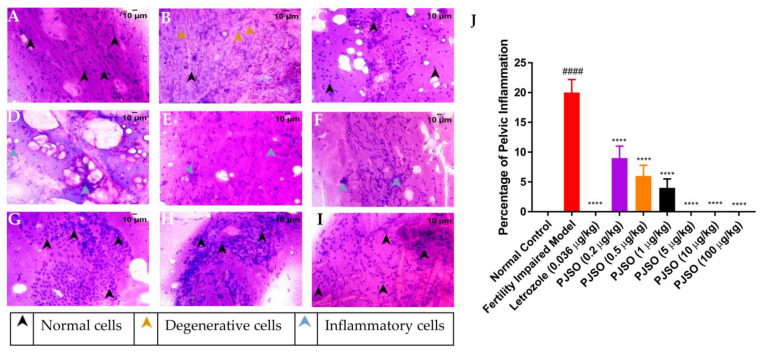
Representative images of Hematoxylin–Eosin-stained cytology smears of ovaries from different study groups used for quantification of pelvic inflammation. Images were taken using a 40× objective. (**A**) Healthy control, (**B**) Fertility-impaired group, (**C**) Fertility-impaired fish treated with 0.036 µg/kg/day letrozole, (**D**) Fertility-impaired females treated with 0.2 µg/kg/day PJSO, (**E**) Treatment group with 0.5 µg/kg/day PJSO, (**F**) Treatment group with 1 µg/kg/day PJSO, (**G**) Treatment group with 5 µg/kg/day PJSO, (**H**) Treatment group with 10 µg/kg/day PJSO, and (**I**) Treatment group with 100 µg/kg/day PJSO. (**J**) Percentage of pelvic inflammation is represented as mean ± SD from 24 individuals and one-way ANOVA followed by Tukey’s multiple comparisons test was used to determine statistical significance. #### represents *p* < 0.0001 compared to the healthy control group, **** represents *p* < 0.0001 compared to the fertility-impaired group.

Ovarian inflammation was quantified using the smear cytology of the ovary from the different groups. The smear was stained with Hematoxylin and Eosin and was observed for the presence of degenerative cells and immune cells, which are used to mark the presence of inflammation. Cells in the control group showed well-defined nuclei and eosinophilic cytoplasm with a high distribution of follicular epithelium and supportive parenchyma (Figure 7A). No immune cells were identified in the cytology smears, suggesting a complete lack of inflammation in the ovary. The fertility-impaired group showed an increased number of degenerative nuclei with loss of eosinophilic staining in the cytoplasm, suggesting inflammation of the ovary (Figure 7B). A decrease in the distribution of the follicular epithelium was also observed, which is one of the reasons for the impaired fertility in this group. The percentage of inflammation calculated from triplicate fields was significantly higher (*p* < 0.0001) in the fertility-impaired group when compared to the healthy control group (Figure 7J). The letrozole treatment group did not show any visible signs of inflammation in the ovary and the cytology showed the presence of yolk vesicles that are slightly eosinophilic, indicating rescue of the phenotype (Figure 7C). Lower dosages of PJSO could not rescue the inflammation completely, but it was significantly less compared to the fertility-impaired group. Cytology smears of treatment groups with 0.2 µg/kg/day (Figure 7D), 0.5 µg/kg/day (Figure 7E), and 1 µg/kg/day (Figure 7F) did not have any degenerative cells but still had the presence of immune cells within the ovary, suggesting that the dosage was not enough to completely rescue the fertility impairment. Higher dosages at 5 µg/kg/day (Figure 7G), 10 µg/kg/day (Figure 7H), and 100 µg/kg/day (Figure 7I) did not show either the degenerative cells or the presence of immune cells, suggesting complete recovery from the inflammation.

### 2.7. Impaired Fertility Rate in Male Zebrafish Was Restored upon Treatment with Putrajeevak Seed Oil

**Figure 8:**
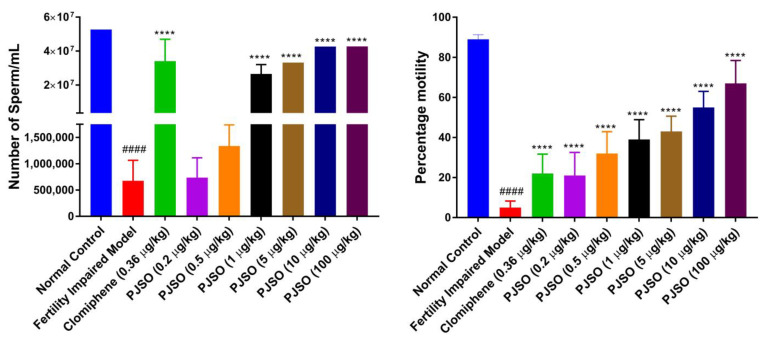
*P. roxburghii* seed oil reverses impaired sperm count and sperm motility in male zebrafish. (**A**) Sperm count per mL determined in different study groups (**B**) Percentage motility of sperm calculated from 1 × 10^8^ sperm. Data are represented as mean ± SD from 24 individuals and one-way ANOVA was used to determine statistical significance. #### represents *p* < 0.0001 compared to the healthy control group, and **** represents *p* < 0.0001 compared to the fertility-impaired group.

The number of sperms produced per mL was measured to determine the functionality of the gonads. Males from the control group had little variation in sperm count between individual fish and had an average count of 5.2 × 10^7^ sperm/mL (Figure 8A). There was a significant reduction (*p* < 0.0001) in the sperm count in the fertility-impaired group with only 1.3% of the healthy control group. Treatment with clomiphene restored the number of sperm to about 65% of the control number, which was highly significant when compared to the fertility-impaired group. Treatment with 0.2 and 0.5 µg/kg/day of PJSO showed only a modest and non-significant increase in the sperm count. Treatment with the other dosages showed a dose-dependent increase in the sperm count, which was statistically significant (*p* < 0.0001) when compared with the fertility-impaired group. The 1 µg/kg/day showed a sperm count 50% of the healthy control, the 5 µg/kg/day group had 63%, and the 10 and 100 µg/kg/day had a sperm count approximately 81% of the healthy control group.

In addition to the sperm count, the motility of the sperm was also analyzed. The motility assay helps in understanding sperm quality, which is essential for successful fertilization. Lower sperm motility may indicate poorly functional sperm and, in turn, reduce the rate of successful formation of an embryo. The fertility-impaired group had a drastically low motility percentage (5%) when compared to the healthy control group (Figure 8B). The clomiphene-treated group showed a significantly higher (*p* < 0.0001) motility percentage compared to the fertility-impaired group but this was still lower at 22%. The PJSO treatment groups showed a dose-dependent increase in the motility percentage, which was statistically significant (*p* < 0.0001) when compared to the fertility-impaired group. However, even with the highest dosage (100 µg/kg/day), the percentage motility was only 67%, unlike the 89% motility seen in the healthy control group.

### 2.8. Treatment with Putrajeevak Seed Oil Improved Testicular Cytological Features

**Figure 9:**
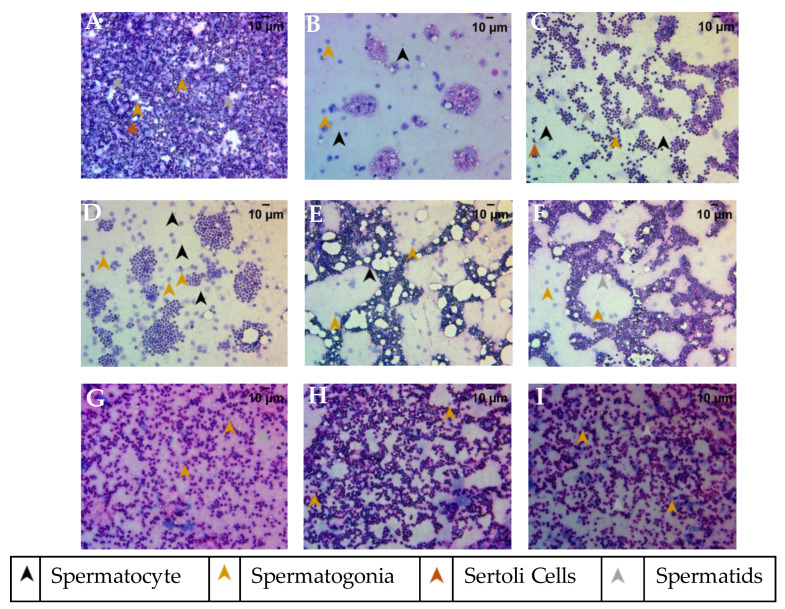
Testicular smears stained with Hematoxylin–Eosin to determine the recovery of impaired fertility in male zebrafish when treated with Putrajeevak seed oil. Images were captured using a 40× objective and representative images from the different study groups are shown. (**A**) Healthy control group, (**B**) Fertility-impaired group, (**C**) Clomiphene (0.36 µg/kg/day) treatment group, (**D**) 0.2 µg/kg/day PJSO treatment group, (**E**) PJSO 0.5 µg/kg/day treatment group, (**F**) PJSO 1 µg/kg/day treatment group, (**G**) 5 µg/kg/day PJSO treatment group, (**H**) 10 µg/kg/day PJSO treatment group, and (**I**) PJSO at 100 µg/kg/day treatment group.

The smear cytology of the testis dissected from the various study groups was stained with Hematoxylin and Eosin to assess the health of the tissue. The cytology from the healthy control group showed a well-defined nuclear morphology with eosinophilic cytoplasm and spermatogonia at various stages of maturity (Figure 9A). The different stages such as spermatogonia, primary spermatocytes, secondary spermatocytes, spermatids, and spermatozoa were identified along with Sertoli cells, in the cytology from the control group. The fertility-impaired group of fish showed a remarkably lower number of spermatozoa, indicating a poor maturation cycle of the sperm (Figure 9B). In addition, a very low number of Sertoli cells and spermatocytes with a higher proportion of spermatogonia were detected. The clomiphene treatment group showed a normal tissue architecture of the testis with spermatogonia at various stages of maturation (Figure 9C). A moderate number of Sertoli cells were also detected. The 0.2 µg/kg/day PJSO treatment group showed a higher proportion of undifferentiated spermatogonia with poor eosinophilic staining, suggesting a functionally compromised gonad (Figure 9D). The groups treated with 0.5 µg/kg/day (Figure 9E) and 1 µg/kg/day PJSO (Figure 9F) showed a very low number of spermatogonia and a higher number of spermatids, suggesting an improvement in the functionality of the tissue. Study groups treated with PJSO at dosages of 5 µg/kg/day (Figure 9G), 10 µg/kg/day (Figure 9H), and 100 µg/kg/day (Figure 9I) showed a higher proportion of spermatids, indicating a complete recovery of the functional gonad. The smear cytology showed the ability of *P. roxburghii* seed oil to restore a fully functional testis with spermatogonia at different maturation stages and reverse the fertility impairment.

## 3. Discussion

This main aim of the study was to understand the role of *P. roxburghii* seed oil in rescuing the impaired fertility phenotype and to correlate the changes seen with the different phytoconstituents identified in the supercritical fluid extract. These studies demonstrated the effect of reversing the N-ethyl-N-nitrosourea (ENU)-induced impaired fertility in a zebrafish model. The use of the supercritical fluid extraction process for pharmacologically active phytocomponents would have ensured accurate and reproducible extraction of constituents that are present in trace amounts.

Putrajeevak seed oil contains a mixture of fatty acids, with unsaturated fatty acids (82.6%) forming the bulk of the oil content. Oleic acid (C18:n9c) made up about half the fatty acid content at 49.2%, with linoleic acid (C18:2n6c) the next most abundant (31.7%). In addition to the fatty acids, β-sitosterol was found to be at 0.98% in PJSO. This phytosterol has a chemical structure similar to cholesterol and is widely distributed in the plant kingdom. Studies in American mink (*Neovison vison*) have shown a slight increase in litter size with dietary β-sitosterol supplementation for a period of 9 months [10]. In this study, the β-sitosterol content in PJSO was found to be 0.98% and even at a maximum dose of 100 µg/kg, the amount of β-sitosterol administered per day was 0.98 ng/kg/day. This was much lower than the maximal dose of nearly 50 mg/kg/day used in American minks.

Studies have shown that only 4% of dietary β-sitosterol is absorbed [11]. Though studies in rats have shown that higher dosages (0.5 to 5 mg/kg in a day) given subcutaneously reduced sperm count and testicular mass [12], studies in fish have shown that β-sitosterol administration caused a decrease in the circulating levels of steroid sex hormones, and increased the production of vitellogenin expression [13,14]. These studies suggest that β-sitosterol may have a rescue effect on infertility at lower dosages.

In our study, fertility in zebrafish was impaired using mutagenesis with N-ethyl-N-nitrosourea (ENU) for a period of 30 days. This causes mutations in both pre- and post-meiotic germ cells, and is an established mode of inducing infertility in both male and female zebrafish [15,16,17]. The accepted protocol for mutagenesis uses a longer duration of exposure (1h) in a smaller number of exposures (4 to 6) at weekly intervals. These protocols had a significant mortality in fish and therefore, the mutagenesis protocol was modified. Using ENU mutagenesis for 15 min per day for 30 days resulted in significantly higher viable fish (98.5%), with 76.1% of these showing fertility impairment as demonstrated by less than 20 embryos being produced. Though several different ratios of male and female fish are used for assessing the rate of fertility [18], we had the best possible rate of positive fertility when a 1:4 (male to female) ratio was used.

In our study, the male zebrafish were also treated with metronidazole and only fish that were able to produce less than 20 fertile eggs when spawned with healthy females were used. Metronidazole leads to cell ablation and can cause complete testicular loss; however, in our study, a full-length treatment regimen was not employed and was paired with ENU mutagenesis to lead to infertility, where the result is a lack of the ability to produce functional semen. Mutagenized females that were able to produce less than 20 viable embryos when spawned with healthy males were chosen for the study. Mutations caused by ENU may lead to further downstream effects resulting in impaired fertility which may not be a direct result of the mutation [7]. The female zebrafish in the fertility-impaired group had an ovary that was smaller in size compared to the healthy control group and had very low follicular count with low egg reserve. Most of the follicles were at either the primary growth or the pre-vitellogenic stages of growth, suggesting that ENU mutagenesis resulted in an ovary with immature eggs. In addition, there was a significantly higher number of follicles that resembled cysts similar to polycystic ovarian syndrome. This suggests that ENU mutagenesis could result in dysfunctional ovaries, not only producing a lower number of mature oocytes but also having an increased number of unviable cysts. In the male zebrafish, this mutagenesis caused a drastic decrease in the sperm count as well as impaired motility of the viable sperm to a large extent. Anatomically, the testis had a significantly lowered number of spermatozoa, indicating a poor maturation cycle.

Letrozole is a known aromatase inhibitor which induces ovulation by reducing estrogens. This, in turn, may increase the production of follicle-stimulating hormone (FSH), thus increasing the production of ovarian follicles [19]. In our study, treatment with letrozole rescued most of the infertility seen in the ENU-treated zebrafish. There was an increase in the fertility rate as well as the fecundity rate, suggesting the increased production of viable larvae from this group. *P. roxburghii* seed oil at human relevant doses restored the fertility rate and the fecundity rate by more than 60% and this was increased to near the levels seen in the healthy controls by treatment at 5× the human dosage and higher. In addition, treatment with PJSO restored the gross anatomy and the cytology of the ovary to levels comparable with that of the healthy individuals.

One of the major causes of female infertility is the presence of cysts in the ovary, similar to that seen in polycystic ovarian syndrome. A cyst is a fluid-filled follicle that cannot develop into a functional egg and letrozole has been used to improve fertility in such cases [20]. In this study, the fertility-impaired group had a significantly higher number of cysts compared to the healthy group and treatment with PJSO reversed cyst formation as well as inflammation seen in the ovary. The presence of cysts was not completely abrogated with letrozole but was significantly reduced, and even the highest dosage of PJSO could not completely reverse the formation of cysts. However, PJSO showed a comparatively better effect on the reduction in cyst formation. In addition to a reduction in the number of cysts, there was a drastic improvement in the number of follicles in the final growth stage of egg development with PJSO. This suggests that *P. roxburghii* seed oil was able to restore ovarian follicle development to near normal levels.

The fatty acid analysis of PJSO showed that oleic acid and linoleic acid form the bulk of the fatty acid content. Experiments using radioisotope-labelled linoleic acid in rats showed a greater utilization of carbon from linoleic acid for cholesterol biosynthesis [21]. The higher levels of linoleic acid in PJSO may cause an increased biosynthesis of cholesterol which can, in turn, increase the levels of glucocorticoids and influence egg development and release [22].

Oligoasthenozoospermia, reduced sperm count and sperm motility, was seen in the zebrafish ENU mutants in this study. Anti-estrogens such as clomiphene have been shown to increase the secretion of gonadotropin-releasing hormone (GnRH) and thus, have been prescribed for empirical treatment of idiopathic male infertility [19,20]. Treatment with PJSO showed a significant increase in the number of spermatids and a reduction in the number of spermatogonia cells. This resulted in an increased number of mature sperm as well as increase in sperm motility, and subsequent increase in the number of positive spawning events. PJSO at human relevant dosage had a slightly lower but non-significant reduction in the number of sperm in comparison to the clomiphene treatment group. Significantly, the percentage of sperm was higher at human relevant dosage of PJSO compared to human relevant dosage of clomiphene, suggesting that PJSO was better at rescuing oligoasthenozoospermia.

## 4. Materials and Methods

### 4.1. Chemicals and Reagents

All chemicals used in the study were of the highest grade and procured from Sigma-Aldrich (St. Louis, MO, USA) unless specified. Phosphate-buffered saline (8 g NaCl, 0.2 g KCl, 1.44 g Na_2_HPO_4_, and 0.24 g KH_2_PO_4_ to a final volume of 1 liter with the pH adjusted to 7.4) and embryo medium (NaCl 294 g, KCl 12.7 g, CaCl_2_·2H_2_O 48.5 g, and MgSO_4_·7H_2_O 81.3 g made up to a liter) were made in house for everyday use.

### 4.2. Isolation of PJSO from P. roxburghii Seeds

Putrajeevak seeds (*Putranjiva roxburghii* Wall.) were procured from Divya Pharmacy, Haridwar, India. The seeds were fully mature and were designated for clinical use. The Putrajeevak seeds were pulverized and made into a fine powder and mixed with food-grade liquid CO_2_. Supercritical carbon dioxide (SC-CO_2_) was used in a supercritical extractor—the SFE 5000 Bio-Botanical Extraction System (Waters Corporation, Milford, MA, USA) [23]. The extractor was equipped with a CO_2_ recycler and the backflow pressure was maintained at 450/80 and 55/4 Bar/°C and a CO_2_ flow rate of 60 g/min over a period of 820 min. Extraction was performed in two stages with a static extraction phase followed by a dynamic extraction phase. The resultant extracted oily substance (batch number D4/CHM/SCFE15710919) was used for the biological experiments and chemical characterizations.

### 4.3. Gas Chromatography for Analysis of Fatty Acid Content

The fatty acid content in PJSO was determined according to the method prescribed by the American Chemist’s Society [24]. Briefly, the supercritical fluid extract, PJSO, was mixed with 0.5 N methanolic NaOH and heated for 10 min to create fat globules. After this, BF_3_-methanol was added to the boiling mixture and was further heated for 2 min. To this, n-Heptane was added and boiled for a minute more. The mixture was removed from the heat, and saturated NaOH solution was added and mixed vigorously. After allowing the mixture to cool for 10 min at room temperature, the top layer of n-Heptane containing fatty acid mixture called the fatty acid methyl ester (FAME) was collected.

Gas chromatography-flame ionized detection (GC-FID) was used to analyze the fatty acid mixture in the FAME using helium as a carrier (GC-2010 Plus gas chromatograph, Shimazdu, Kyoto, Japan). A standard mix for C4-C24 FAME containing a mixture of 37 FAMEs (Sigma-Aldrich, St. Louis, MO, USA) was used as a standard to ascertain the identities of the fatty acids present in the PJSO.

### 4.4. Identification of β-Sitosterol in PJSO Using HPTLC

#### 4.4.1. Sample Solution Preparation

The supercritical fluid extract of Putrajeevak seeds, PJSO (600 mg), was dissolved in 5 mL chloroform. The sample was sonicated for 20 min on ice (Fisherbrand Model 505, Hampton, NH, USA) to obtain a clear solution and then centrifuged at 8000 rpm for 5 min at room temperature to further clarify. The supernatant was transferred to a volumetric flask and the volume was made up to 10 mL with chloroform.

#### 4.4.2. Preparation of Standard Solution

Stock solution of β-sitosterol (Natural Remedies, Bangalore, Karnataka, India) was prepared by dissolving 10 mg in 1 mL of methanol. Working standards were further diluted in methanol to obtain solutions of 400 to 1200 µg/mL, which were then used for identification and quantification of β-sitosterol in PSJO.

#### 4.4.3. HPTLC for Quantification of β-Sitosterol in PJSO

Standard β-sitosterol at different concentrations and known volumes of PSJO was spotted on aluminum-backed silica gel 60 F_254_ plates (Merck Millipore KGaA, Darmstadt, Germany). Chloroform:methanol (9:1) was used as a mobile phase and anisaldehyde sulfuric acid reagent was used for derivatization. Quantitation was by densitometry after scanning the derivatized samples at 450 nm. The concentration of β-sitosterol in the sample was estimated against a linearity curve from duplicate loadings.

### 4.5. Animal Ethics

All animal handling protocols were followed under the Committee for the Purpose of Control and Supervision of Experiments on Animals (CPCSEA), Govt of India, for Good Animal Practices. Approved protocols from the Institutional Animal Ethics committee (vide protocol number: 216/Go092019/IAEC) were followed throughout the animal study.

### 4.6. Zebrafish Husbandry

Groups of zebrafish (*Danio rerio*) Wild-Type AB strain at 1.2 to 1.5 years of age, from the same spawning, were grouped together at 24 fish per group in polypropylene tanks with 2 liters of water per fish with a light/dark cycle of 14/10 h. The water temperature was maintained at 27 ± 1 °C with constant aeration and water circulation to remove wastes and excess food particles. Water pH was checked every day and was adjusted accordingly if needed. Water exchange was performed every week by removing half of the existing water and replenishing it with aged de-chlorinated water.

### 4.7. Induction of Fertility-Impaired Zebrafish

Fish in the study groups were treated with N-ethyl-N-nitrosourea (ENU) to induce infertility. The accepted mode of induction using ENU requires an hour long treatment with 3 mM ENU each week for a period of 4 to 6 weeks [15,16,17]. However, in our hands, this method resulted in an unacceptable level of mortality and, therefore, this method was modified to not only produce fertility-impaired mutants but also to reduce mortality. Briefly, adult fish, both male and female, were exposed to 5 mM ENU in water for 15 min each day for 30 days; they were then transferred to normal housing water. In the case of male zebrafish, the fish were further treated with 100 mM metronidazole by water dissolution for a further 20 days. Metronidazole causes cell ablation, leading to a lower number of functional cells and, in combination with ENU mutagenesis, reversible loss of semen production [25,26].

Groups of 6 male and female fish were randomly selected and were dissected to isolate the testes and ovaries for anatomical and pathological observation to confirm smaller sized glands and reduced spermatogonia in males and a lower number of ovarian follicles in female fish.

Post mutagenesis, the fish were maintained in normal husbandry conditions for 14 days to allow the fish to overcome the stress. The female fish were allowed to spawn with healthy males (1:4 female to male ratio) to ensure a more positive fertility rate. Though lower ratios were also used, it was observed that a 1:4 (female to male ratio) improved the rate of fertility [18]. In the case of mutant males, they were allowed to spawn with healthy females (1:1 ratio). The spawning happened between 6.30 to 7.30 in the morning under controlled conditions at 28 ± 1 °C. Purposive sampling was employed to select mutant fish for the study; only females with an embryo count less than 20 and males that resulted in less than 20 embryos with healthy females were selected for the study.

### 4.8. Study Design

Once the study population was selected, they were acclimatized to the study husbandry condition for 7 days. Fish were fed with normal Tetrabit flakes (a complete pet food for tropical fish from Tetra GmbH, Herrenteich, Germany) until the start of the experimental period. The fish were fed in a 24 h feeding cycle throughout the study period. A schematic of the study design is shown in Figure 2.

#### 4.8.1. Dosing and Preparation of Feed

The seeds of *P. roxburghii* are directly consumed and the daily recommended quantity according to Ayurvedic texts is 2 to 3 g per day. For the calculation of human dosage for the supercritical fluid extracted oil, an average of 2.5 g was taken. The yield of oil was determined over multiple extractions and an average yield of 3% was taken. The equivalent dosage of the oil, calculated from the recommended weight of seeds to be consumed and the average yield of oil, was determined as ~75 mg/day. For the study, the human dosage of PJSO was taken as 70 mg/day. During the experimental period, the fish were fed with either the reference drugs or with different dosages of the *P. roxburghii* seed oil (0.2×, 0.5×, 1×, 5×, 10×, and 100× compared to relevant human doses).

Different dosages of the compound were calculated for a known volume of feed. The study compound was mixed with the feed pellets and finely ground using a mortar and pestle. The feed mixture was extruded into pellets of standard size weighing 4 mg per pellet. The dosages were the same for both genders, and the translational doses for the different study groups were calculated according to the guidelines of USFDA [27] and are given in Table 4. Clomiphene was used as a comparator for male infertility, while letrozole was used in the female infertility study.

#### 4.8.2. Endpoints with Mutant Female Zebrafish

The dosing was for a period of 14 days, after which the fish were allowed to spawn with healthy males on the 15th day. The fertility rate, conception rate, and fecundity rate were assessed during the spawning, with a ratio of 1 mutant female to 4 healthy males. After the completion of a second cycle of ovulation, the ovary was dissected and the follicle count (ovarian reserve of eggs) was estimated. In a parallel experiment, after 14 days of dosing, the ovary was dissected on the 15th day and the overall morphology was assessed for the quantification of cysts. After this, the cytological smear was stained with Hematoxylin–Eosin and the percentage of inflammation was assessed.

#### 4.8.3. Endpoints with Mutant Male Zebrafish

After the acclimatization period of a week, the fish were fed with either clomiphene as a comparator or Putrajeevak seed oil at the same dosages as with the female fish for a period of 14 days. The fertility rate was estimated in spawning experiments with wild-type female fish (1:1 ratio) in standard breeding conditions on the 15th day and the sperm count per mL, motility of the sperm, and testicular cytology smear were assessed.

### 4.9. Dissection

Fish were euthanized with 2 to 4 °C water [28]. The fish was dissected through an incision in the ventral side from the gills to the vent. The ovary and the testis were isolated by cutting open the viscera with a dissection knife. The ovary was identified by its large size, lobed morphology, cream color, and the presence of eggs within. The testis was identified as a semi-transparent, lobed structure and a large size.

#### Hematoxylin–Eosin Staining

Cytology smears were prepared from either the ovary or the testis; these were used to replicate the clinical diagnostic criteria as close as possible, where biopsies are used to diagnose fertility impairment [29,30]. The smear was fixed on a glass slide and stained with Hematoxylin for 2 min followed by Eosin for a further 2 min. Excess stain was washed multiple times in PBS at pH 7.4 and viewed under 40× bright field objective using a Labomed l× 400 microscope (Labomed, Los Angeles, CA, USA). Images were captured using the image viewer software. Triplicate fields were randomly chosen and the cells per field were quantified using Qupath software [31].

### 4.10. Assessment of Study Endpoints in Female Zebrafish

#### 4.10.1. Fertility Rate

The success of mating was assessed by the presence of embryos irrespective of their viability. The fertility rate was calculated based on the total number of positive spawning events that led to the formation of an embryo to the total number of breeding pairs.

#### 4.10.2. Rate of Conception

Conception affirms that both fertilization has taken place as well as the presence of highly confluent embryos that are viable. The conception rate was calculated as the number of highly confluent viable embryos per breeding event to the total number of embryos fertilized eggs.

#### 4.10.3. Fecundity Rate

Embryos collected from the breeding tank were transferred to 30 mL embryo medium for estimation of the number of viable larvae obtained from the embryos. The fecundity rate was calculated as the number of larval survivors from the viable embryos from each spawning event at 10 dpf.

#### 4.10.4. Follicle Count—Ovarian Egg Reserve

The ovary of an adult zebrafish is thick and lobular anatomically, and contains non-synchronously developing oocytes [32]. The four different stages of oocyte development before the formation of a mature egg (Full growth) are classified as Stage I—Primary growth (PG); Stage II—Pre-vitellogenic; Stage III—Early vitellogenic stage (EV); Stage IV—Late vitellogenic stage.

The ovary was isolated from the dissected fish and was washed twice in PBS. The ovary was dispersed on a glass slide manually to release the follicles; the matured follicles were identified as yolk-filled, dense, morphological structures. The number of oocytes at different developmental stages was counted using a micro-columned slide to estimate the ovarian reserve of eggs. The yolk-filled follicles were counted using the 40× objective of a stereomicroscope (Labomed, Los Angeles, CA, USA).

#### 4.10.5. Ovarian Cyst Quantification

Cysts on the ovary were identified as a dark mass of cells that are irregularly shaped under 40× objective of a stereomicroscope. The presence of cysts is considered as a sign of immature follicles, leading to increased infertility in female zebrafish.

#### 4.10.6. Quantification of Pelvic Inflammation

To study inflammation, the ovary was isolated from euthanized fish and prepared for smear cytology. The smear was stained with Hematoxylin–Eosin and the presence of cellular debris along with increased presence of neutrophils and lymphocytes was recorded.

### 4.11. Assessment of Study Endpoints in Male Zebrafish

#### 4.11.1. Sperm Count in Male Zebrafish

A capillary tube capable of holding 2 µL volume was used for sperm collection. The fish were anesthetized in water at 17 °C until the operculum movement came to a stop. They were dried gently on a paper towel and placed in a holding chamber with the ventral side up. The cloacal region was gently wiped clean to ensure that all the moisture was removed as this could prematurely activate the sperm. The capillary tube with an attached aspirator was positioned at the cloaca and the sperm was collected by gently massaging the region with padded forceps to produce the ejaculate. This was collected into the capillary tube till the desired mark and then drained onto a glass slide for microscopy. Images were captured in three random fields and the total number of sperm was counted using Qupath software.

#### 4.11.2. Motility of the Sperm

The sperm was freshly collected as mentioned previously and the motility was assessed according to the protocol devised by Selvaraj et al. [33] with modifications. Briefly, 2.2 µL of the ejaculate was diluted to 20 µL using Hank’s Balanced Salt Solution (HBSS) at an osmolality of 300 mOsmol/kg, and was used immediately. The sample was centrifuged at 428× *g* for 5 min and resuspended in 1 mL of HBSS. After the number of sperm was counted, they were centrifuged again at 285× *g* and finally, resuspended at a concentration of approximately 1 × 10^8^ sperm per µL of HBSS. Following this, 1 µL of sperm was transferred to a clean glass slide and the motility of the sperm was recorded using a 10× objective.

The raw video files were transferred to Image J and the scale was set as per the video resolution. The video was processed using Z project and was run through the wrMTrck plugin to track the number of moving objects [34]. The cumulative number of moving objects was considered as the progressive motility of spermatozoa.

### 4.12. Data Analysis and Statistics

All data points are expressed as mean ± standard deviation (SD) of observations. Statistical analysis was performed using GraphPad Prism 7.04 (GraphPad Software Inc., San Diego, CA, USA). One-way ANOVA followed by Tukey’s multiple comparisons test was used to compute the significance of the data. In the analysis, the fertility-impaired group was compared to the healthy control group, while the reference drug and PJSO treatment groups were compared to the fertility-impaired group. *p*-values less than 0.5 were considered significant.

## 5. Conclusions

Fatty acid analysis of *P. roxburghii* seed oil revealed oleic acid and linoleic acid as the major constituents. Trace amounts of β-sitosterol were also identified using HPTLC. ENU mutagenesis was successfully used to establish a fertility-impaired zebrafish model in this study. Putrajeevak seed oil has been shown to restore the ovarian reserve of eggs and the production of mature eggs, and restore fertility rate in a female infertility model. In addition, PJSO enhanced recovery of the follicular structures within the ovary as well as caused a reduction in the number of cysts. These results were comparable to the effect of letrozole in restoring the fertility in ENU-mutagenized female fish. PJSO also restored the sperm count and sperm motility in ENU-mutagenized male zebrafish similar to that seen with clomiphene. Overall, these results suggest that *P. roxburghii* seed oil administration is useful in reversing the impaired fertility in both the genders.

## Figures and Tables

**Table 1:** Fatty acid constituents of the supercritical fluid extract of Putrajeevak seeds and their quantity as identified by gas chromatography-flame ionized detection. The chemical structures were sourced from the Royal Society of Chemistry website (www.chemspider.com (accessed on 19 January 2021)).

Name	Molecular Formula	Molecular Weight	Chemical Structure	Content in PJSO (%)
Palmitic Acid	C_16_H_32_O_2_	256.4	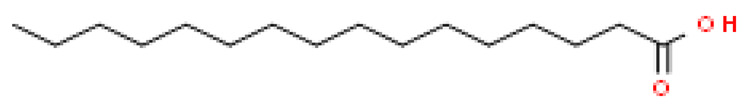	8.67
Stearic Acid	C_18_H_36_O_2_	284.48	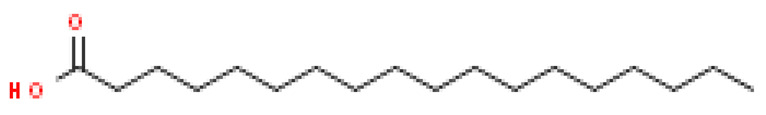	8.67
Oleic Acid	C_18_H_34_O_2_	282.5	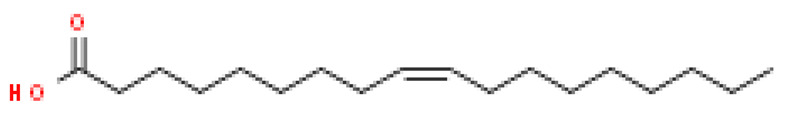	49.29
Linoleic Acid	C_18_H_32_O_2_	280.4	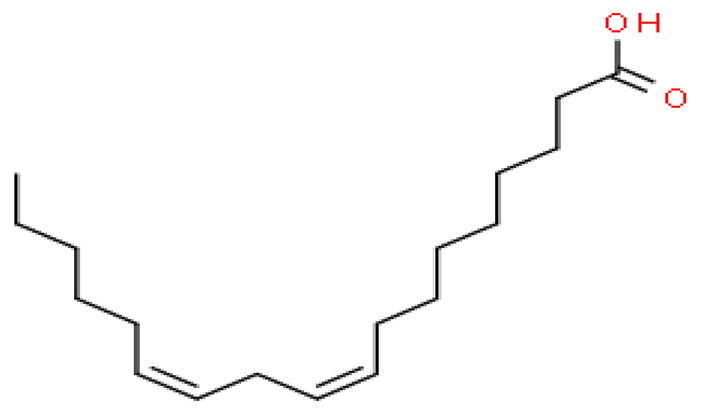	31.74
Linolenic Acid	C_18_H_30_O_2_	278.4	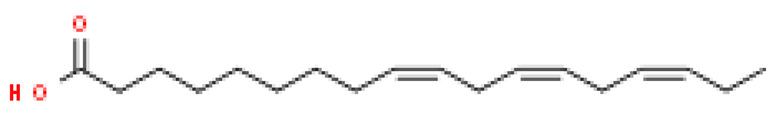	0.81
Cis-11,14-Eicosenoic Acid	C_20_H_36_O_2_	308.5	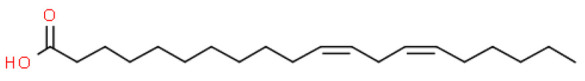	0.79

**Table 2:** Percentage of positive spawning events in the different study groups. The formation of an embryo irrespective of the health of the embryo was considered as a positive spawning event (*n* = 24, *p* < 0.0001).

Study Groups	Percentage of Positive Breeding	
	Female	Male
Control	100	100
Fertility-Impaired Model	83.3 ± 37.2 ^####^	75 ± 43.3 ^####^
Reference Standard	100	87.5 ± 33.0 ****
PJSO (0.2 µg/kg)	100	79.1 ± 40.6 ****
PJSO (0.5 µg/kg)	100	91.6 ± 27.6 ****
PJSO (1 µg/kg)	100	95.8 ± 19.9 ****
PJSO (5 µg/kg)	100	100
PJSO (10 µg/kg)	100	100
PJSO (100 µg/kg)	100	100

#### represents significance compared to the healthy control group; **** represents significance compared to the fertility-impaired group.

**Table 3:** Ovarian reserve of eggs determined by the number of ovarian follicles at different stages of development. Data are represented as mean ± SD of 24 individuals and one-way ANOVA followed by Tukey’s multiple comparison test was used to arrive at statistical significance; ns represents no significance, * represents *p* < 0.5 compared to the fertility-impaired group, #### represents *p* < 0.0001 compared to the healthy control, **** represents *p* < 0.0001 compared to the fertility-impaired group.

Study Groups	Follicle Count—Ovarian Reserve of Eggs					
	Primary Growth (PG)	Pre-Vitellogenic (PV)	Early Vitellogenic (EV)	Mid-Vitellogenic (MV)	Final Growth (FG)	Total
Control	114 ± 8.2	296 ± 8.1	311 ± 8.3	109 ± 7.8	483 ± 7.6	1313 ± 16.3
Fertility-Impaired Model	97 ± 6.8 ^####^	79 ± 8.9 ^####^	16 ± 6.8 ^####^	9 ± 7.6 ^####^	12 ± 6 ^####^	213 ± 15.3 ^####^
Letrozole (0.036 µg/kg)	137 ± 7 ****	256 ± 7.7 ****	140 ± 7.4 ****	96 ± 8.4 ****	356 ± 7.6 ****	985 ± 18.0 ****
PJSO (0.2 µg/kg)	101 ± 7.7	81 ± 7.4	23 ± 8 *	9 ± 6.2	98 ± 7.2 ****	311 ± 16.5 ****
PJSO (0.5 µg/kg)	110 ± 6.8 ****	89 ± 8.4 ****	51 ± 6.8 ****	16 ± 8.6 *	168 ± 6 ****	434 ± 16.2 ****
PJSO (1 µg/kg)	216 ± 7.4 ****	129 ± 8.2 ****	108 ± 7.5 ****	41 ± 7.7 ****	227 ± 8 ****	721 ± 14.4 ****
PJSO (5 µg/kg)	233 ± 9.2 ****	229 ± 7.9 ****	141 ± 6.9 ****	52 ± 6.9 ****	301 ± 8.5 ****	956 ± 17.7 ****
PJSO (10 µg/kg)	350 ± 6.7 ****	94 ± 7.8 ****	60 ± 7.8 ****	56 ± 7.5 ****	411 ± 8 ****	972 ± 19.4 ****
PJSO (100 µg/kg)	353 ± 3 ****	96 ± 3.5 ****	64 ± 3 ****	59 ± 4.2 ****	434 ± 3.3 ****	1005 ± 8.4 ****

**Table 4:** Translational dose of standard drug and Putrajeevak seed oil derived from human equivalent dose per day.

Study Groups	Standard Drug			Putrajeevak Seed Oil (PJSO)						
	Drug	Human Dose	Translational Dose for Zebrafish	Human Dose	Translational Dose for Zebrafish					
					0.2×	0.5×	1×	5×	10×	100×
Male	Clomiphene	25 mg/day	0.36 µg/kg	70 mg/day	0.2 µg/kg	0.5 µg/kg	1 µg/kg	5 µg/kg	10 µg/kg	100 µg/kg
Female	Letrozole	2.5 mg/day	0.036 µg/kg	70 mg/day	0.2 µg/kg	0.5 µg/kg	1 µg/kg	5 µg/kg	10 µg/kg	100 µg/kg

## Data Availability

The data presented in this study can be made available upon request from the scientific community on the discretion of the corresponding author.

## Sample Availability

Samples of the compounds are available from the author on request for suitable academic research.
